# PReS-FINAL-2031: Hyaluronic acid is a marker of active arthritis, but not systemic inflammation in systemic juvenile idiopathic arthritis

**DOI:** 10.1186/1546-0096-11-S2-P44

**Published:** 2013-12-05

**Authors:** B Hugle, M Olsson, N Fischer, M Herrmann, K Lindblad-Toh, JP Haas

**Affiliations:** 1 German Center for Pediatric and Adolescent Rheumatology, Garmisch-Partenkirchen, Germany; 2 Medical Biochemistry and Microbiology, Science for Life Laboratory, Uppsala University, Uppsala, Sweden; 3 Internal Medicine, University Hospital Erlangen, Erlangen, Germany

## Introduction

Systemic juvenile idiopathic arthritis (JIA) is a subtype of childhood arthritis associated with significant systemic inflammation as well as arthritis. Hyaluronic acid as a constituent of connective tissue has previously been described as a marker for arthritis in JIA in an Asian population. Mutations in hyaluronic acid synthase 2 leading to accumulation of hyaluronic acid have been found to be associated with a periodic fever syndrome in Shar-Pei dogs. Hyaluronic acid has therefore been suspected as a risk factor for systemic inflammation in humans.

## Objectives

To investigate the correlation of hyaluronic acid levels with clinical manifestations and markers of inflammation in patients with systemic juvenile idiopathic arthritis.

## Methods

Patient sera and clinical data were acquired from the AIDnet database, a German database and biobank where samples from patients with inflammatory syndromes including periodic fevers syndromes and systemic JIA are collected prospectively. A single center sample of all patients diagnosed with systemic JIA was obtained, and hyaluronic acid levels were determined from frozen sera samples. Demographic data, data on medication and laboratory data on inflammatory parameters (C-reactive protein, ESR, S100A8/A9 and S100A12) from the time points the serum samples were obtained were extracted from the AIDnet database. A retrospective chart survey was used to determine systemic inflammatory activity and arthritis activity. Patients were deemed to have systemic activity if they had fever and at least one other symptom according to the ILAR criteria at time of obtaining the sample, and were deemed to have active arthritis if they had at least one swollen joint or tenderness and limitation of motion at time of obtaining the sample. Univariate analysis was performed using descriptive statistics, Student's T-test and the Pearson correlation coefficient.

## Results

25 patients were included in the study, of which 13 had active arthritis and 3 active systemic symptoms at time of sampling. 48% were treated with prednisone, 76% with a DMARD and 72% with a biologic drug at time of sampling. Patients with systemic symptoms had a mean level of hyaluronic acid of 61.0 μg/l, compared to 134.5 μg/l in patients with no systemic symptoms (p = 0.330). However, patients with active arthritis had a mean level of hyaluronic acid of 180.5 μg/l, compared to 48.1 mg/l in patients without active arthritis (p = 0.002). No significant correlations were found between levels of hyaluronic acid and levels of CRP, ESR, S100A8/A9 and S100A12.

## Conclusion

In this Caucasian population, hyaluronic acid levels are correlated with clinical arthritis, but not with systemic activity or inflammatory parameters in systemic juvenile idiopathic arthritis. Hyaluronic acid does not appear to be a driver of systemic inflammation in sJIA.

## Disclosure of interest

None declared.

